# Tissue-Specific DNA Methylation Changes in CD8^+^ T Cells During Chronic Simian Immunodeficiency Virus Infection of Infant Rhesus Macaques

**DOI:** 10.3390/v16121839

**Published:** 2024-11-27

**Authors:** Mukta Nag, Jonathan E. Fogle, Santhoshan Pillay, Gregory Q. Del Prete, Kristina De Paris

**Affiliations:** 1 AIDS and Cancer Virus Program, Frederick National Laboratory for Cancer Research, Frederick, MD 21702, USA; 2 Department of Microbiology and Immunology, University of North Carolina, Chapel Hill, NC 27599, USAkristina_abel@med.unc.edu (K.D.P.); 3 Boehringer Ingelheim Animal Health, Athens, GA 30601, USA

**Keywords:** CD8^+^ T cells, DNA methylation, pediatric HIV infection, pediatric SIV infection, infant rhesus macaque model, tissue-specific methylation, tissue-specific cytokine regulation

## Abstract

Robust CD8^+^ T cell responses are critical for the control of HIV infection in both adults and children. Our understanding of the mechanisms driving these responses is based largely on studies of cells circulating in peripheral blood in adults, but the regulation of CD8^+^ T cell responses in tissue sites is poorly understood, particularly in pediatric infections. DNA methylation is an epigenetic modification that regulates gene transcription. Hypermethylated gene promoters are associated with transcriptional silencing and, conversely, hypomethylated promoters indicate gene activation. In this study, we evaluated DNA methylation signatures of CD8^+^ T cells isolated from several different anatomic compartments during pediatric AIDS-virus infection by utilizing the SIV_mac239/251_ infected infant rhesus macaque model. We performed a stepwise methylation analysis starting with total cellular DNA, to immunomodulatory cytokine promoters, to specific CpG sites within the cytokine promoters in CD8^+^ T cells isolated from peripheral blood, lymph nodes, and intestinal tissue during the chronic phase of infection. Tissue-specific methylation patterns were determined for transcriptionally active promoters of key immunomodulatory cytokines: interferon gamma (IFNγ), interleukin-2 (IL-2), and tumor necrosis factor alpha (TNFα). In this study, we observed tissue-specific differences in CD8^+^ T cell modulation by DNA methylation in SIV-infected infant macaques, highlighting the importance of evaluating cells from both blood and tissues to obtain a complete picture of CD8^+^ T cell regulation during pediatric HIV infection.

## 1. Introduction

CD8^+^ T cells are key mediators of viremia control during HIV/SIV infections. The involvement of CD8^+^ T cells in viral control is evidenced by the concurrent emergence of virus-specific CD8^+^ T cells during peak viremia resolution in the acute phase of infection, the increase in viremia that follows experimental depletion of CD8^+^ T cells in SIV-infected rhesus macaques in both acute and chronic phases of infection, and the selection of viral CTL-escape mutations in response to the immunological pressure exerted by CD8^+^ T cells [1,2,3,4,5,6,7]. In addition, specific MHC class I alleles, HLA B*27 and B*57, are associated with elite control of HIV viral replication, with HLA B*27- and B*57-positive elite controllers shown to possess high frequencies of virus-specific CD8^+^ T cells restricted by these class I alleles [8,9,10,11]. Akin to humans, elite control of SIV replication in rhesus macaques is associated with MHC class I alleles Mamu B*08 and B*17 [12]. While most of these studies are based on analysis of CD8^+^ T cells isolated from peripheral blood, CD8^+^ T cells exhibit tissue-specific viral control mechanisms [13]. Tissue-resident CD8^+^ T cells in lymphoid tissue and gastrointestinal mucosa are phenotypically and functionally distinct from peripheral blood CD8^+^ T cells as they, unlike blood-derived CD8^+^ T cells, can contribute to the control of viral infection via non-cytolytic mechanisms in addition to cytolytic mechanisms [14,15,16,17,18]. Immunomodulatory cytokines such as interferon gamma (IFNγ), interleukin-2 (IL-2), and tumor necrosis factor alpha (TNFα) modulate the overall quality of T cell responses during HIV/SIV infection. These cytokines regulate T cell proliferation, survival, antiviral and cytolytic functions among other supporting roles for CD4^+^ and CD8^+^ T cells [19]. However, persistent antigen exposure and heightened immune activation during HIV/SIV infection results in progressive loss of function and proliferative capacity of CD8^+^ T cells leading to a pool of exhausted CD8^+^ T cells during chronic infection across all tissue sites [20,21].

While CD8^+^ T cell-mediated viral control of HIV has been well studied in adults, our understanding of the underlying mechanisms by which it does so in pediatric HIV infection, particularly in difficult-to-access tissue sites of viral replication, such as gut and lymph nodes, is severely limited. Dynamic changes in the developing immune system continue into the post-natal period in infants where the immune cells are qualitatively and quantitatively different from adults [22,23,24,25]. Infants with HIV often have persistent high levels of viremia with elevated CD8^+^ T cell activation in the chronic phase of infection compared with adults despite comparable levels of CD8^+^ T cell proliferation early during infection as measured in blood [26,27]. The lack of CTL escape mutants in circulating virus in children reflects the weaker immunological selection pressure exerted by virus-specific CD8^+^ T cells compared to adults, even when the CTL responses are restricted by protective MHC alleles such as HLA-B*57 [28,29]. Immature adaptive immune responses in infants and key differences in HIV/SIV pathogenesis compared to adults necessitate the elucidation of molecular mechanisms regulating CD8^+^ T cell responses across different tissue sites during pediatric HIV/SIV infection.

Epigenetic modifications including DNA methylation regulate CD8^+^ T cell differentiation, effector cell expansion, effector function, and exhaustion in lentiviral infections [30,31,32,33,34,35]. DNA methylation is a reversible epigenetic modification where a methyl group is added to the 5′-C of the cytosine residue in the context of CG, CHH, or CHG within a DNA sequence by DNA methyltransferases [36]. Changes in CpG methylation levels directly impact gene transcription. An increase in CpG DNA methylation at a gene promoter region results in gene repression, while a demethylated promoter is indicative of active gene expression [36]. In adult chronic HIV infection, hypomethylation at the Programmed Death 1 (PD1) promoter has been associated with a concomitant increase in PD1 protein expression in virus-specific compared to naïve CD8^+^ T cells, suggesting a role for DNA methylation in CD8^+^ T cell exhaustion [35]. However, in pediatric HIV/SIV infection, DNA methylation changes mediating CD8^+^ T cell regulation at different tissue sites remain unexplored.

As a first step towards understanding the mechanism of DNA methylation-based regulation of CD8^+^ T cells during pediatric HIV/SIV infection in both blood and tissues, in this study, we utilized specimens from SIV_mac239/251_ infected infant rhesus macaques to characterize tissue-specific CpG methylation signatures at the IFNγ, IL-2, and TNFα promoters in CD8^+^ T cells during chronic phase of pediatric SIV infection. We performed a systematic assessment of the methylation status of CD8^+^ T cells from peripheral blood, lymph nodes, and intestinal tissues at the global DNA, cytokine promoter-wide, and CpG site-specific level in infant rhesus macaques to identify differentially methylated CpG sites during SIV infection. Our study represents the first known methylation analysis of CD8^+^ T cells isolated from often inaccessible tissues such as lymph nodes and gut from infant rhesus macaques during SIV infection. The results from this study may inform a detailed investigation of the role of DNA methylation in modulating CD8^+^ T cell effector responses at various tissue sites during pediatric HIV/SIV infection.

## 2. Methods

**Animals, sample collection, and processing:** This study was conducted with archived, cryopreserved peripheral blood mononuclear cells (PBMCs) or mononuclear cell suspensions (MNCs) from axillary/inguinal/mesenteric lymph nodes (LNs), or intestinal (ileum/ colon) tissues of SIV-infected infant rhesus macaques included in prior studies [37,38,39]. At the time of those studies, all animals were nursery-reared and pair-housed at the UC Davis California National Primate Research Center. Study protocols and sample collections had been approved by the UC Davis Animal Care and Use Committee and were conducted in adherence to the guidelines of the Guide for the Care and Use of Laboratory Animals (*National Research Council. 2011. Guide for the care and use of laboratory animals, 8th ed. National Academies Press, Washington, DC, USA*). Age, SIV status, and SIV plasma viremia of rhesus macaques from which samples were obtained are listed in Table 1.

**Cell stimulation and CD8^+^ T cell isolation:** Freshly thawed PBMCs or tissue MNCs were resuspended at 1.5 × 10^6^ cells/mL of RPMI 1640 supplemented with 10% fetal bovine serum (FBS), 1% penicillin-streptomycin, and 1% L-glutamine. Cells were stimulated with 5 μg/mL of concanavalin A (Con A) (Sigma, St. Louis, MO, USA) at 37 °C for 2 h to promote non-antigen-specific proliferation and cytokine production [40]. Stimulated cells were washed with PBS (Gibco, Gaithersburg, MD, USA) at 500× *g* for 7 min and resuspended at 10^7^ cells/mL in PBS+ 2% FBS (Serum Source International, Charlotte, NC, USA) + 1 mM EDTA (Thermo Fisher Scientific, Waltham, MA, USA). CD8^+^ T cells were isolated using a custom-made Easy Sep™ Negative-Selection NHP CD8^+^ T cell isolation kit with D-magnetic beads (Stem Cell, Vancouver, BC, USA). In brief, 52 μl/mL of CD8^+^ T cell negative selection antibody cocktail and 50 μl/mL of γδ-CD8^+^ T cell-depletion antibody were added to 10^7^ cells and incubated for 10 min at RT with constant shaking. Magnetic beads in suspension at 150 μl/mL of cells were added and incubated at RT for 5 min. CD8^+^ T cells were then negatively selected according to the StemCell protocol; this step was repeated twice to ensure maximum recovery. The purity of CD8^+^ T cells, as determined by flow cytometry, was >92% and the cell viability was >90% (See Supplementary Material S1).

**DNA extraction and global methylation ELISA:** Genomic DNA was extracted from Con A stimulated PBMCs and tissue-derived CD8^+^ T cells using the QIAamp DNA Mini Kit (Qiagen, Hilden, Germany) and quantified using a NanoDrop Lite Spectrophotometer (Thermo Scientific, Waltham, MA, USA). Global DNA methylation was measured using the MethylFlash Global DNA Methylation 5-mc ELISA kit (Epigentek, Farmingdale, NY, USA) according to the manufacturer’s instructions. This kit allows for colorimetric quantification of global DNA methylation by specifically measuring 5-methylcytosine (5-mc) levels in input genomic DNA.

**Targeted Bisulfite Sequencing of Cytokine Promoter Regions**

*PCR amplification of bisulfite reduced genomic DNA:* Genomic DNA (input: 250 ng) isolated from PBMCs and tissue-derived CD8^+^ T cells was bisulfite converted using the EZ DNA Methylation-Gold Kit (Zymo Research, Irvine, CA, USA). Primers for bisulfite-reduced targets within the rhesus macaque IL-2, IFNγ, and TNFα promoter regions were designed using the Zymo Research Bisulfite Primer Seeker (Zymo Research) as listed in Table 2 (Supplementary Material S2). These selected regions of cytokine promoters had multiple predicted transcription factor binding sites as determined by the web-based tool PROMO which uses the transcription factor binding site database TRANSFAC version 6.4 for its predictions [41,42]. The PCR reaction cocktail consisted of 5 µL of template DNA, 12.5 µL KAPA HiFi Uracil + Readymix (Kapa Biosystems, Wilmington, MA, USA), 0.4 µM (1 µL) forward primer, 0.4 µM (1 µL) reverse primer and 5.5 µL of nuclease-free water. PCR amplification was conducted under the following cycling conditions: hot start enzyme activation at 95 °C for 5 min, 40 cycles of denaturation at 98 °C for 20 s, annealing at 60 °C for 15 s, and 72 °C for 1 min, followed by 72 °C for 1 min and a final 4 °C hold step. PCR products were sequenced to confirm the promoter sequences (Genewiz, Germantown, MD, USA).

*Library preparation and sequencing:* Libraries of the promoter amplicons were prepared using the Nextera XT DNA Library Prep Kit and Nextera XT Index Kit (Illumina Inc., San Diego, CA, USA; protocol February 2018, v03). DNA was quantified on a Qubit^®^ 3.0 Fluorometer using a Qubit dsDNA High Sensitivity Kit (Life Technologies, Carlsbad, CA, USA). Briefly, 5 µL of DNA (at a concentration of 0.2 ng/µL) was fragmented using 5 µL Amplicon Tagment Mix with 10 µL of Tagment DNA buffer (Illumina Inc.). Tagmentation reactions were performed by incubation at 55 °C for 5 min followed by the addition of 5 µL Neutralize Tagment Buffer. The tagmented DNA (25 µL) was used as the template in a 50 µL adapter ligating PCR (15 cycles). Amplified DNA was purified using 1.8× of the volume of AMPure XP beads (Beckman Coulter Inc., Brea, CA, USA). The fragment size distribution of the tagmented and adapter-ligated DNA was analyzed utilizing an Experion™ Automated Electrophoresis System (Bio-Rad, Hercules, CA, USA). Barcoded DNA libraries were pooled and sequenced using a paired-end, 2 × 150 read length MiSeq platform at the High Throughput Sequencing Facility (HTSF) Core at the University of North Carolina at Chapel Hill. Raw reads were re-allocated to each DNA sample’s unique Nextera barcode during analysis. 

*CpG methylation analysis:* Fastq files from HTSF were analyzed using the CLC Genome Workbench software (version 11, CLC Bio, Qiagen). Files were first merged for forward and reverse reads to the appropriate barcoded samples. Paired PBMC reads were individually aligned to reference sequences acquired from the NCBI database (Supplementary Material S2). The reference sequences were bisulfite-converted by the CLC Genome Workbench program. Reads from tissue CD8^+^ T cells were grouped by SIV status, creating a consensus sequence that represented the alignment of all reads selected against the specified reference gene sequence based on the chosen mapping parameters (non-directional, 0.8 similarity fraction, non-specific matches mapped randomly). The resulting sequences were then used to determine the percent CpG methylation of the experimental sample compared to the reference sequence. DNA methylation was assessed across the whole amplicon and at specific CpG sites within the amplicon.

**Statistical analysis:** Statistical analysis was performed using GraphPad Prism software version 10.0. Paired PBMC samples were compared using a two-tailed paired *t*-test while the unpaired tissue sample data were compared using the Mann–Whitney test. *p* values of *p* < 0.05 were considered statistically significant. 

## 3. Results

### 3.1. Genomic DNA Methylation in PBMC, Lymph Node, and Intestinal CD8^+^ T Cells During SIV Infection

**Figure 1:**
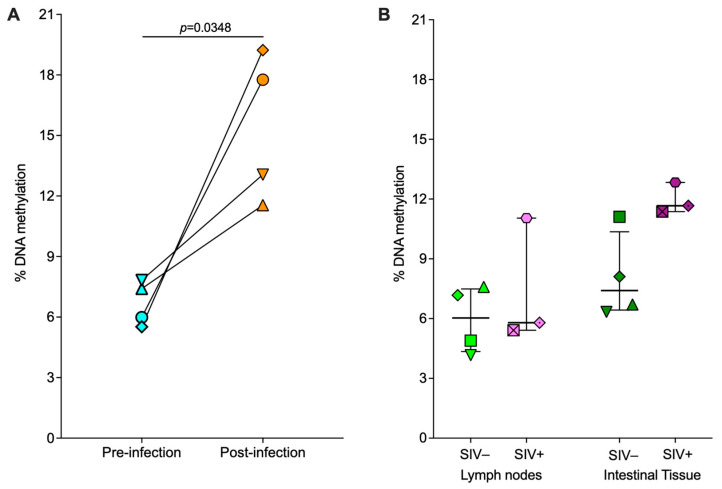
Global DNA methylation levels of CD8^+^ T cells derived from PBMCs, lymph nodes, and intestinal tissues of SIV uninfected and infected rhesus macaques. Methylation levels were quantitated for genomic DNA isolated from bulk CD8^+^ T cells derived from paired PBMCs pre- and post-SIV infection (**A**), and from lymph nodes and intestinal tissues of unpaired SIV-uninfected and SIV-infected rhesus macaques (**B**). Panel (**A**): Change in DNA methylation post (orange symbols)- versus pre (blue symbols)- infection within the same animal (n = 4). Data represent the average methylation levels from 3 technical replicates per animal, with each animal being represented by a unique symbol. Statistical significance was determined by a two-tailed paired *t*-test in GraphPad Prism. Panel (**B**): Mean DNA methylation levels ± standard deviation in lymph nodes (light-colored symbols) and intestinal (dark-colored symbols) tissues of 4 SIV uninfected (green and olive symbols) and 3 SIV infected (pink and purple symbols) rhesus macaques. Note that these animals are distinct from the animals shown in (**A**). Each SIV uninfected and infected animal in (**B**) is represented by a unique symbol, with corresponding symbols in lymph nodes or intestinal tissue for the same animal being light or dark-colored, respectively. PBMC samples from SIV-infected animals were collected between 12 and 13 weeks post-infection, whereas lymph node and intestinal tissue samples of SIV-infected animals were collected between 4 and 9 weeks post-infection.

The overall degree of genomic DNA methylation provides insight into the global epigenetic regulatory status of cells of interest [43]. Therefore, we first quantitated the levels of 5-methylcytosine (5-mc) in genomic DNA from bulk CD8^+^ T cells isolated from peripheral blood (PBMCs) and from mononuclear cell suspensions derived from lymph nodes and intestine. Measured global DNA methylation levels were higher in PBMC-derived CD8^+^ T cells collected following SIV infection compared with matched pre-infection samples collected from the same four animals (Figure 1A).

Unlike the PBMC samples, longitudinal pre- and post-infection samples for lymph node and intestinal tissue from the same animals were not available. Instead, we compared paired lymph node and intestinal CD8^+^ T cells from four SIV-naïve animals with three SIV-infected animals for tissue-specific assays as listed in Table 1. Mean global DNA methylation percentages were higher in both lymph node (7.4%) and intestinal (11.9%) CD8^+^ T cells in the three SIV-infected animals compared to CD8^+^ T cells of lymph nodes (5.9%) and intestinal tissues (8.0%) of SIV-naïve animals, but the differences were not statistically significant (Figure 1B). However, within the SIV-naïve group, there was a trend towards higher baseline global DNA methylation levels in the intestinal compared to lymph node CD8^+^ T cells, and a similar difference was observed within the SIV-infected group (Figure 1B). Overall, despite the lack of statistical significance in this comparison between limited numbers of tissue samples, these data suggest that global DNA methylation levels in SIV-infected versus uninfected infant rhesus macaque CD8^+^ T cells may vary across different anatomical compartments.

### 3.2. Methylation of the IFNγ, IL-2, and TNFα Cytokine Promoters in CD8^+^ T Cells of SIV-Naïve and SIV-Infected Infant Rhesus Macaques

IFNγ, IL-2, and TNFα are essential cytokines for CD8^+^ T cell function and immune regulation during SIV infection [44,45]. We next analyzed DNA methylation patterns at the promoters of these immunomodulatory cytokines in CD8^+^ T cells during SIV infection in infant macaques. CpG dinucleotides prone to methylation changes are clustered in CpG islands (CGI), which are enriched in gene promoters and overlap with transcription start sites (TSS) [46]. Hypermethylation of the CGIs is frequently associated with transcriptional repression [43]. Therefore, we quantitated promoter-wide methylation changes in regions up to 1000 bp upstream of the TSS for IFNγ, IL-2, and TNFα (Table 3). These regions of cytokine promoters were selected because they are enriched in multiple predicted transcription factor binding sites critical for downstream gene regulation (Table 4).

**Figure 2:**
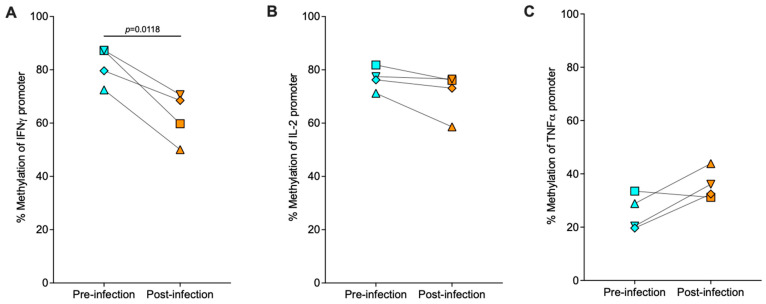
**Promoter-wide CpG methylation changes in IFNɣ, IL-2, and TNF⍺ promoters of peripheral blood CD8^+^ T cells post-versus pre-SIV infection**. CpG methylation changes in the region of interest within IFNɣ (**A**), IL-2 (**B**), and TNF⍺ (**C**) gene promoters were quantitated pre- (blue symbols) and post- (orange symbols) SIV infection in paired peripheral blood CD8^+^ T cells. The percentage of methylation of each animal was calculated as (methylated reads/total reads) × 100. Each matched symbol represents a unique animal, n = 4. Statistical significance was assessed by a two-tailed paired *t*-test in GraphPad Prism. PBMC samples from SIV-infected animals were collected between 12 and 13 weeks post-infection.

Figure 2 shows the cytokine promoter-wide methylation changes recorded in PBMC-derived CD8^+^ T cells during SIV infection compared to prior to infection. Methylation at the IFNγ promoter declined (*p* = 0.0118) in all 4 animals post- compared with pre-infection (Figure 2A). At the IL-2 promoter, methylation levels decreased in 3 of 4 animals post-SIV infection compared with pre-infection, although this difference was not significant (*p* = 0.1133) likely because methylation levels remained unchanged in 1 of 4 animals (Figure 2B). In contrast, at the TNFα promoter, there was a trend towards increased methylation post- compared to pre-infection (*p* = 0.0942); however, this increase was only observed in 3 of 4 animals while the levels decreased in 1 of 4 animals post- compared with pre-infection (Figure 2C). The hypomethylated state of the IFNγ and IL-2 promoters in 4 of 4 and 3 of 4 animals, respectively, post-SIV infection compared to pre-infection in PBMC-derived CD8^+^ T cells indicated active gene transcription based on the known dogma of permissiveness of hypomethylated promoter for downstream gene activation, whereas the increase in methylation at the TNFα promoter in 3 of 4 animals post-infection was expected to be associated with repression of gene transcription compared to pre-infection. These data suggested differential regulation of distinct cytokine promoters in peripheral blood CD8^+^ T cells during SIV infection, encouraging us to perform a more specific and nuanced, base-level methylation change comparison.

**Figure 3:**
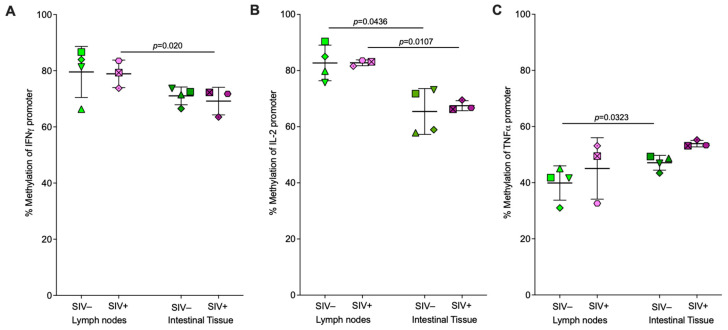
**Promoter-wide methylation levels at the IFNɣ, IL-2, and TNF⍺ promoters in lymph node and intestinal tissue derived CD8^+^ T cells from SIV uninfected and infected rhesus macaques**. Methylation levels of select region of the IFNɣ (**A**), IL-2 (**B**), and TNF⍺ (**C**) gene promoters were compared between lymph node (light-colored symbols) and intestinal tissue (dark-colored symbols) derived CD8^+^ T cells within SIV-uninfected (green and olive symbols, n = 4) and SIV-infected (pink and purple symbols, n = 3) animals. Data represent the mean and standard deviation in methylation of all the animals per group, with each animal being represented by unique symbols. Statistical differences between mean methylation percentages of the same CD8^+^ T cell cytokine promoter across different tissues in the same animal were calculated by a two-tailed paired *t*-test in GraphPad Prism. No statistically significant differences were observed in comparisons based on infection status, i.e., the methylation of the same cytokine promoter was compared between CD8^+^ T cells of the same tissue type in SIV-uninfected compared to SIV-infected animals (significance calculated by Mann–Whitney test in GraphPad Prism, see Table 5 for results). Lymph node and intestinal tissue samples of SIV-infected animals were collected between 4 and 9 weeks post-infection.

Unlike the changes observed at each cytokine promoter in PBMC-derived CD8^+^ T cells, there were no differences between SIV-infected and SIV-uninfected animals in the measured methylation levels at the cytokine promoters in CD8^+^ T cells derived from lymph node or intestinal tissues (Table 5, Figure 3).

However, the cytokine promoters in CD8^+^ T cells were differentially methylated across the two tissues, lymph node, and intestine, within the same animal. In SIV-uninfected animals, differences in methylation were observed for the IL-2 and the TNFα promoters. The IL-2 promoter of SIV-uninfected animals was hypomethylated in intestinal (Mean% ± standard deviation: 65.4% ± 8.1) compared to lymph node (82.7% ± 6.3) derived CD8^+^ T cells (Figure 3B). Although there was a trend towards reduced mean methylation in the IFNγ promoter of intestinal CD8^+^ T cells (71.0% ± 3.1) versus lymph node CD8^+^ T cells (79.5% ± 9.0), this difference was not significant as one animal (upwards triangle symbol) had higher methylation at the intestinal compared to the lymph node site (Figure 3A). In contrast, the TNFα promoter exhibited higher methylation in the intestinal (47.0% ± 2.6) compared to the lymph node (39.8% ± 6.1) CD8^+^ T cells in the same animal (Figure 3C). In SIV-infected animals, both the IFNγ and IL-2 promoters had lower methylation in the intestinal (IFNγ: 69.1% ± 4.9; IL-2: 67.4% ± 1.7) compared to the lymph node (IFNγ: 78.8% ± 4.8, IL-2: 82.7% ± 1.0) CD8^+^ T cells (Figure 3A,B). A trend towards higher CpG methylation in the TNFα promoter of intestinal CD8^+^ T cells (53.9% ± 1.1) versus lymph node CD8^+^ T cells (45.0% ± 10.9) in SIV-infected animals was observed, however, was not significant (Figure 3C).

### 3.3. CpG Site-Specific Methylation at CD8^+^ T Cell Cytokine Promoters of Different Tissues in SIV-Infected Compared to Uninfected Infant Rhesus Macaques

**Figure 4:**
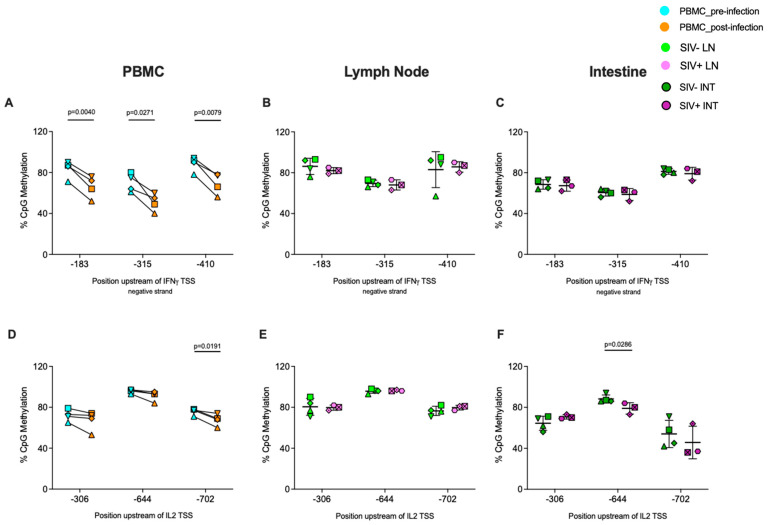
**Site-specific methylation of CpG residues within the IFNɣ and IL-2 promoter regions of CD8^+^ T cells isolated from PBMC, lymph nodes, and intestinal tissues of SIV-uninfected and SIV-infected infant rhesus macaques.** Methylation levels were measured for each CpG residue within the selected region of the IFNɣ (**A**–**C**) and IL-2 (**D**–**F**) promoters of CD8^+^ T cells derived from different tissues. Panel (**A**): The change in methylation post- (orange symbols) versus pre- (blue symbols) SIV infection at each CpG site within the same animal (each symbol represents a unique animal, n = 4). Statistical significance was assessed by a two-tailed paired *t*-test in GraphPad Prism. Panels (**B**,**C**): Methylation levels of each CpG site in SIV uninfected (green and olive symbols, n = 4) and infected (pink and purple symbols, n = 3) macaque CD8^+^ T cells derived from lymph nodes (light-colored symbols) (**B**) or intestinal tissues (dark-colored symbols) (**C**), respectively. Data represent the mean and standard deviation in methylation of all the animals per group, with each animal being represented by unique symbols in panels (**B**,**C**). Statistically significant differences were calculated by the Mann–Whitney test in GraphPad Prism for panels (**B**,**C**). Panels (**D**–**F**) are analogous to panels (**A**–**C**) but illustrate methylation at CpG sites in the IL-2 promoter. PBMC samples from SIV-infected animals were collected between 12 and 13 weeks post-infection, whereas lymph node and intestinal tissue samples of SIV-infected animals were collected between 4 and 9 weeks post-infection.

After quantitating the overall methylation status of cytokine promoters, we sought to evaluate methylation signatures at specific CpG sites within these promoters across different tissue samples during SIV infection. The region of interest for the IFNγ promoter had three CpG sites at positions 183, 315, and 410 base pairs upstream of the IFNγ gene TSS as listed in Table 3 (Supplementary Material S2). Methylation changes were only detected in the DNA-negative strand. A reduction in methylation was observed at all three sites in peripheral blood CD8^+^ T cells in all four animals post- compared to pre-SIV infection (Figure 4A). In lymph node and intestinal CD8^+^ T cells, no difference was observed in CpG methylation at the IFNγ promoter in SIV-infected compared to uninfected animals (Figure 4B,C).

The IL-2 promoter region of interest had three CpG sites at 306, 644, and 702 base pairs upstream of the *IL-2* gene TSS as listed in Table 3 (Supplementary Material S2). In peripheral blood, CD8^+^ T cells, methylation decreased at position −306 in 3 of 4 animals and at positions −702 and −644 in 4 of 4 infant rhesus macaques post-SIV infection compared to pre-infection, although the reduction was only significant at position −702 (Figure 4D). In lymph node-derived CD8^+^ T cells, no methylation differences were observed at any of the CpG sites in SIV-infected versus uninfected animals (Figure 4E). Methylation was lower, however, at one of the three CpG sites, site −644, in the IL-2 promoter of intestinal CD8^+^ T cells of SIV-infected animals compared to SIV-naïve animals (Figure 4F).

**Figure 5:**
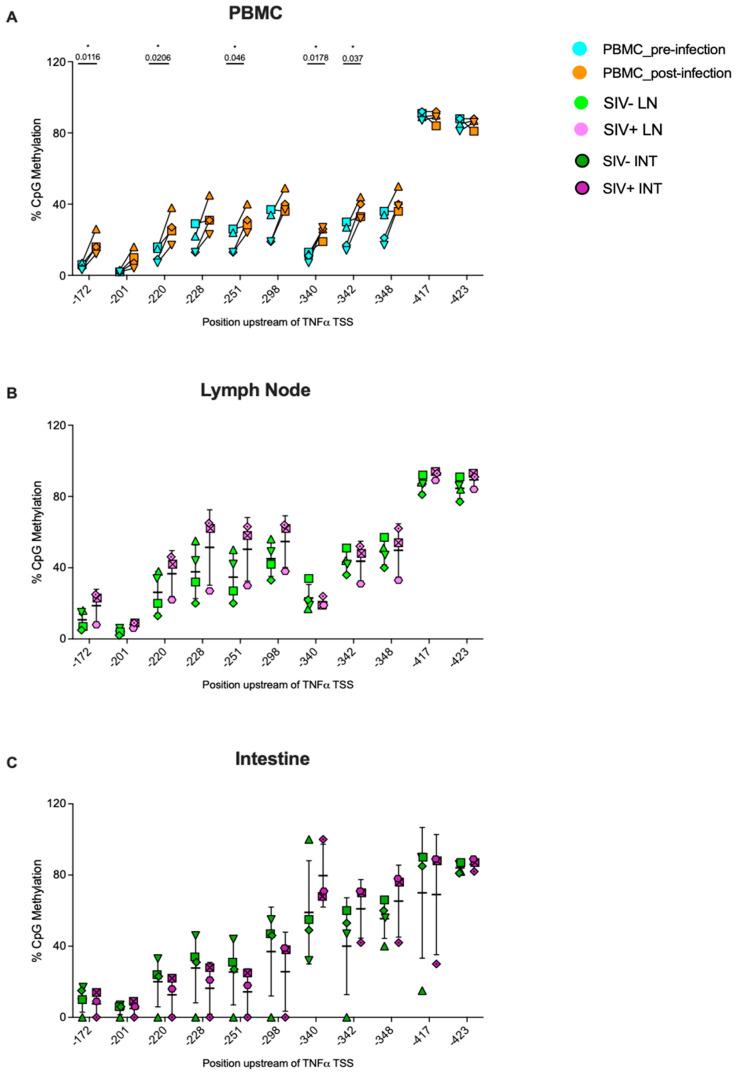
**Site-specific methylation levels of CpG residues within the TNF⍺ promoter region of CD8^+^ T cells isolated from PBMCs, lymph nodes, and intestinal tissues of SIV-uninfected and SIV-infected infant rhesus macaques.** Methylation levels were measured for each CpG residue within the selected region of the TNF⍺ (**A**–**C**) promoter of CD8^+^ T cells derived from different tissues. Panel (**A**): The change in methylation post- (orange symbols) versus pre- (blue symbols) SIV infection at each CpG site within the same animal (each symbol represents a unique animal, n = 4). Statistical significance was assessed by a two-tailed paired *t*-test in GraphPad Prism. “*” denotes statistical significance at *p* value <0.05 with exact *p* values stated on the graph. Panels (**B**,**C**): Methylation levels of each CpG site in SIV uninfected (green and olive symbols, n = 4) and infected (pink and purple symbols, n = 3) macaque CD8^+^ T cells derived from lymph nodes (light-colored symbols) (**B**) or intestinal tissues (dark-colored symbols) (**C**), respectively. Data represent the mean and standard deviation in methylation of all the animals per group, with each animal being represented by unique symbols in panels (**B**,**C**). No statistically significant differences were observed in panels (**B**,**C**) by the Mann–Whitney test in GraphPad Prism. PBMC samples from SIV-infected animals were collected between 12 and 13 weeks post-infection, whereas lymph node and intestinal tissue samples of SIV-infected animals were collected between 4 and 9 weeks post-infection.

The TNFα promoter region assessed in this study contained 11 CpG sites as listed in Table 3 (Supplementary Material S2). At 5 of 11 sites (positions, −172, −220, −251, −340, and −342), there was an increase in methylation after SIV infection compared to prior to infection in peripheral blood CD8^+^ T cells (Figure 5A). SIV-infected animals had high animal-to-animal variability in the methylation levels at specific CpG sites of the TNFα promoter in lymph node and intestinal CD8^+^ T cells. While 2 of 3 SIV-infected animals had higher methylation at sites −172, −220, −228, −251, and −298 of the TNFα promoter in lymph node CD8^+^ T cells compared to the 4 SIV-naïve animals, methylation in the 3rd SIV-infected animal was relatively low and comparable to SIV-naïve animals (Figure 5B). In intestinal CD8^+^ T cells, however, some of the same CpG sites exhibited reduced methylation in SIV-infected compared to SIV-naïve animals (Figure 5C). Overall, though, SIV status did not significantly affect site-specific TNFα promoter methylation in lymph node or intestinal CD8^+^ T cells.

### 3.4. Differences in CpG Site-Specific Methylation Patterns at Each Cytokine Promoter in Lymph Node and Intestinal Tissue CD8^+^ T Cells

**Figure 6:**
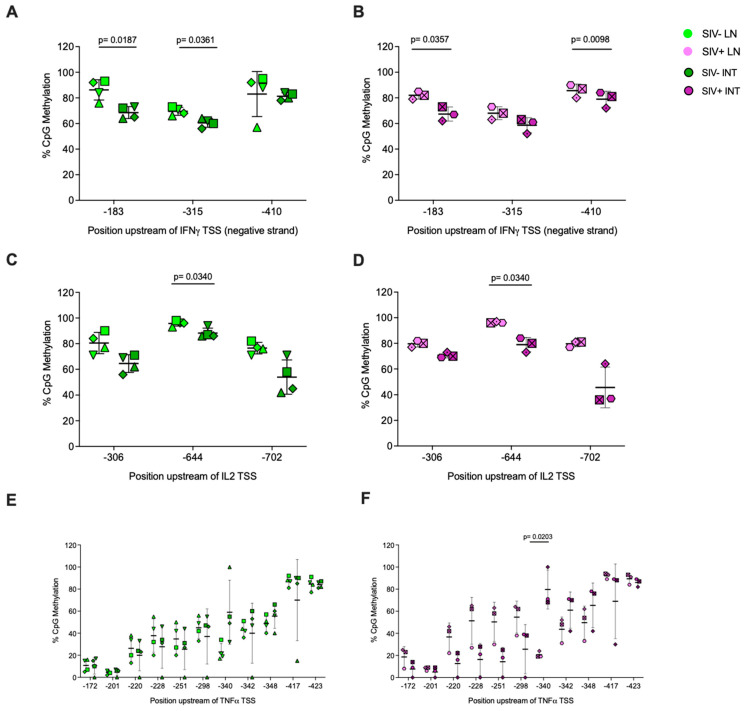
**Tissue-specific differences in CpG site-specific methylation levels within cytokine promoter regions of CD8^+^ T cells isolated from lymph nodes or intestinal tissues in SIV-uninfected and SIV-infected rhesus macaques.** Methylation levels were measured for each CpG residue within the selected region of the IFNɣ (**A**,**B**), IL-2 (**C**,**D**), or TNF⍺ (**E**,**F**) promoters of CD8^+^ T cells derived from lymph nodes (light-colored symbols) and intestinal tissues (dark-colored symbols), respectively. Each symbol represents an individual animal with tissues from SIV-uninfected (green and olive symbols, n = 4) or SIV-infected (pink and purple symbols, n = 3) animals. The data represent the mean and standard deviation in methylation of all the animals per group, with each animal being represented by unique symbols. Statistical significance was determined by a two-tailed paired *t*-test in GraphPad Prism. Lymph node and intestinal tissue samples of SIV-infected animals were collected between 4 and 9 weeks post-infection.

Because we had observed differences in cytokine promoter-wide methylation between CD8^+^ T cells isolated from lymph nodes or intestinal tissues of the same animal, we also tested for tissue-specific methylation differences at specific CpG sites for each cytokine promoter within the same animal in both SIV uninfected and infected groups. There was a trend towards lower CpG methylation in intestinal compared to lymph node CD8^+^ T cells from both SIV-naïve and SIV-infected animals at all three sites in the IFNγ and IL-2 promoters (Figure 6A–D). Methylation was significantly lower in intestinal compared to lymph node CD8^+^ T cells of SIV-naïve animals at CpG sites −183 and −315 at the IFNγ promoter (Figure 6A). While the difference in methylation at CpG site −183 was also observed in SIV-infected animals, differences at site −315 were diminished, and in addition, site −410 exhibited lower methylation in intestinal compared to lymph node CD8^+^ T cells of SIV-infected animals (Figure 6B). In the IL-2 promoter, the difference was significant for the −644 site in both SIV-naïve and infected animals (Figure 6C,D). In the TNFα promoter, 9 of 11 CpG sites trended towards equal or lower methylation in intestinal compared to lymph node CD8^+^ T cells of SIV-naïve animals, but CpG sites −340 and −348 exhibited an opposite trend with higher methylation in intestinal versus lymph node CD8^+^ T cells (Figure 6E). In SIV-infected animals, methylation at site −340 was significantly higher in intestinal compared to lymph node CD8^+^ T cells, and a trend towards higher methylation in intestinal CD8^+^ T cells was also observed at sites −342 and −348 (Figure 6F).

Overall, both promoter-wide and CpG-site specific methylation in CD8^+^ T cells of SIV-naïve animals were higher at the IFNγ and IL-2 promoter compared to the TNFα promoter at all tissue sites. During SIV infection, promoter-wide and CpG-site specific methylation decreased at the IFNγ and IL-2 promoters but increased at the TNFα promoter in peripheral blood CD8^+^ T cells (Figure 2, Figure 4A,D and Figure 5A). Independent of SIV status, promoter-wide and site-specific CpG methylation at the IFNγ and IL-2 promoters trend higher in lymph node compared to intestinal CD8^+^ T cells, whereas distinct CpG sites within the TNFα promoter may express higher or lower relative methylation in lymph node versus intestinal CD8^+^ T cells (Figure 3 and Figure 6).

## 4. Discussion

CD8^+^ T cells are key players in the control of HIV/SIV infection. However, persistently high levels of antigen exposure during untreated chronic HIV infection leads to the progressive loss of CD8^+^ T cell function [8,27], motivating an effort to define molecular mechanisms regulating CD8^+^ T cell responses within various tissues, particularly in the setting of understudied pediatric HIV/SIV infection. In young infants that acquire HIV perinatally or by breastfeeding, CD8^+^ T cell function may further be impacted by dynamic post-natal development processes involving immune system cells [22]. Mechanisms regulating CD8^+^ T cell responses are poorly understood in pediatric infections, in part due to the strikingly low number of pediatric or adolescent population-specific studies [47] and small sample volumes that limit the extent and diversity of analyses that can be performed on the available samples.

The current study is a first step towards defining the role of DNA methylation, an epigenetic modification regulating gene expression, in the regulation of CD8^+^ T cells in various anatomic compartments during pediatric AIDS virus infection. Utilizing the infant rhesus macaque SIV_mac239/251_ infection model, we describe the tissue-specific methylation signatures of CD8^+^ T cells during chronic SIV infection by performing a stepwise methylation analysis of total genomic DNA, immunomodulatory cytokine promoters, and CpG sites within those cytokine promoters across peripheral blood, lymph nodes, and intestinal tissue. We focused our analysis on the modulation of IFNγ, IL-2, and TNFα cytokine promoters as the autocrine effect of these cytokines is one of the mechanisms required for modulating proliferation and antiviral function of CD8^+^ T cells in response to HIV/SIV infection [8,48].

Among the cytokine promoters evaluated, the most substantial methylation differences at each tissue site during SIV infection compared to infection-naïve animals were observed at the IFNγ promoter of CD8^+^ T cells. The higher permissiveness of the IFNγ promoter for active gene expression inferred by the hypomethylation observed in PBMC CD8^+^ T cells post-SIV infection compared to pre-infection (Figure 2A) and intestinal compared to lymph node CD8^+^ T cells in SIV-infected animals (Figure 3A) likely represents one of the mechanisms underlying the higher IFNγ mRNA [49] and cytokine levels in plasma that have been described during HIV-1 infection [50,51]. Site-specific methylation assessment of CpG sites within the IFNγ promoter provided a more detailed picture of methylation alterations in CD8^+^ T cells from different tissues during SIV infection. The reduction in methylation at all 3 CpG sites post-SIV-infection compared to pre-infection in PBMC CD8^+^ T cells (Figure 4A) and the hypomethylation of site −410 in SIV-infected animals exclusively in intestinal compared to lymph node CD8^+^ T cells (Figure 6B) suggest that these alterations are induced by SIV infection. The DNA sequence containing these differentially methylated CpG sites included the predicted binding sites for various transcription factors involved in T cell activation and transcriptional regulation such as Nuclear Factor of Activated T cells-1 (NFAT-1), NFAT-2, T-cell Factor (TCF) isoforms, Lymphoid Enhancer Factor (LEF-1), and NF-KB (Nuclear Factor-kappa B) (Table 4). It is likely that CpG site −410, which coincides with the predicted binding site for NFAT-1, NFAT-2, c-Ets-1, and NF-KB (Table 4) plays an important functional and regulatory role in IFNγ gene expression during SIV infection in PBMCs and intestinal tissues, although this would need to be tested further.

In contrast to the IFNγ promoter, we did not measure substantial methylation alterations at the IL-2 promoter associated with SIV infection. Studies utilizing the Jurkat T cell line and murine CD4^+^ T cells have shown that the IL-2 promoter and enhancer region is prone to methylation changes in response to stimulation, with methylation changes in a small region of the IL-2 promoter associated with measurable transcriptional changes [52,53,54]. Previous studies have also shown that stimulation-driven methylation changes in CpG sites proximal to TSS have a direct impact on *IL-2* gene transcription levels in murine and human cord blood CD4^+^ T cells [53]. In our study, we therefore evaluated methylation changes in a region within the IL-2 promoter immediately proximal, up to 600 bp upstream, of the TSS. Though we did not observe significant methylation changes at the IL-2 promoter in PBMC CD8^+^ T cells post-SIV infection compared to pre-infection (Figure 2B and found lower methylation in intestinal CD8^+^ T cells compared to lymph nodes in both SIV-naïve and infected animals (Figure 3B), it remains possible that the promoter region critical for IL-2 transcriptional control during SIV infection may be different in infant rhesus macaques from the one assessed in our study, which was based on the results of previous human and murine studies. Other regions of the IL-2 promoter and IL-2 regulatory regions need to be examined to conclusively evaluate whether methylation changes occur at the IL-2 promoter in response to SIV infection in infant macaques. However, the selected IL-2 promoter region contains predicted binding sites for multiple inducible and constitutive transcription factors, including NFAT1/2 that are regulated based on cell activation status and TCF, Oct-1, and forkhead box protein P3 (Foxp3) that are constitutively expressed (Table 4). CpG site −702, a predicted Foxp3 binding site, showed reduced methylation in all animals post-infection versus pre-infection in PBMC-CD8^+^ T cells (Figure 4D), while CpG site −644, a predicted C/EBPβ binding site, was hypomethylated in intestinal CD8^+^ T cells in SIV-infected animals compared to SIV-uninfected animals (Figure 4F). The proximity of these differentially methylated CpG sites to key regulatory transcription factor binding sites suggests they may be essential for IL-2 transcriptional regulation. The demethylation of CpG site −702 post-SIV-infection versus pre-infection at the IL-2 promoter in PBMC—CD8^+^ T cells was in contrast with a previous report where none of the IL-2 promoter CpG sites were differentially methylated in PBMC- CD8^+^ T cells in HIV-1 non-controllers compared to controllers and uninfected individuals [55]. This discrepancy could reflect differences in CD8^+^ T cell regulation by SIV versus HIV-1, the differences in host species, differential modulation of CD8^+^ T cells in infants versus adults, or a combination of all three.

DNA methylation patterns at the TNFα promoter during SIV infection differed from the IL-2 and IFNγ promoters. At the TNFα promoter, we observed a trend towards increased promoter-wide methylation in PBMC CD8^+^ T cells post-SIV infection compared to pre-infection (Figure 2C) and a trend towards higher methylation in both intestinal and lymph node CD8^+^ T cells in SIV-infected compared to uninfected animals (Figure 3C), suggestive of gene repression during chronic SIV infection. Five of 11 CpG sites assessed in the TNFα promoter were hypermethylated in PBMC CD8^+^ T cells post-SIV infection compared to pre-infection (Figure 5A), of which only 1 CpG site at position -340 was significantly hypermethylated in intestinal compared to lymph node CD8^+^ T cells in SIV-infected animals (Figure 6F). These differentially methylated CpG sites coincided with key regulatory transcription factor binding sites such as C/EBPβ, Foxp3, and YY1 (Table 4) supporting their potential in TNFα regulation during SIV infection. The data show that the regulation of TNFα is different from IFNγ and IL-2 likely due to its inhibitory and activation roles during HIV-1/SIV infection and its pleiotropic effects on various cell types [56], whereas IFNγ and IL-2 are classically associated with CD8^+^ T cell effector function. One can speculate that the higher number of CpG sites in the selected region of TNFα promoter (11 sites) compared to three sites each in the IFNγ and IL-2 promoters likely results in differential downstream gene regulation of TNFα compared to IFNγ and IL-2, although this would require direct testing. Overall, our cytokine-specific data suggest that in CD8^+^ T cells from chronic SIV_mac239/251_ infection of infant rhesus macaques, IFNγ and IL-2 promoters are poised for active transcription with lower methylation levels, which contrasts with the expectation from functionally dysregulated CD8^+^ T cells associated with chronic SIV infection.

We also evaluated and compared tissue-specific methylation differences in CD8^+^ T cells during chronic SIV infection. In addition to assessing peripheral blood-derived CD8^+^ T cells, we also defined methylation signatures of CD8^+^ T cells from key tissue sites of viral replication that are difficult to access in human pediatric studies. In peripheral blood, the data clearly indicated that global DNA methylation of CD8^+^ T cells increased after SIV infection (Figure 1A). However, when we focused on specific cytokine promoters in PBMC-derived CD8^+^ T cells, cytokine-specific differences emerged where there was a significant decrease in promoter methylation for the IFNγ promoter, a trend towards decreasing methylation for the IL-2 promoter, but an increase in TNFα promoter methylation post SIV-infection compared to prior to infection (Figure 2). These trends were supported by the examination of methylation of the distinct CpG sites within each cytokine promoter. Our data were not in agreement with a study reported by Nakayama-Hosoya et al. where no difference was observed in the methylation levels of IL-2, IFNγ, and TNFα promoters in PBMC-derived CD8^+^ T cells isolated from HIV-1 non-controllers compared to controllers and uninfected individuals, perhaps due to species-specific and/or virus-specific differences between the two studies [55]. It is also possible that the differences in methylation levels in PBMCs are more easily visualized when comparing longitudinal post versus pre-infection samples from the same animal as opposed to in a cross-sectional analysis as reported by Nakayama-Hosoya et al. Compared to peripheral blood, HIV/SIV viral burden and associated immunological features are different in lymph node and intestinal tissues [15,57]. As the main sites of viral replication, viral burden is higher in intestinal and lymph nodes harboring a higher number of viral RNA^+^ T cells compared to peripheral blood [58]. Our assessment of SIV infection-associated DNA methylation differences in CD8^+^ T cells from lymph nodes and intestinal tissues compared to uninfected animals was less conclusive than the peripheral blood dataset likely due to the absence of longitudinal tissue samples post SIV- infection compared to prior to infection. Unlike the peripheral blood dataset, where we had paired samples for comparison, tissue CD8^+^ T cells of 4 SIV-naïve animals were compared to the same tissue type but obtained from 3 different SIV-infected animals. Therefore, while we also observed a trend towards higher global DNA methylation in lymph node and intestinal CD8^+^ T cells from SIV-infected compared to SIV-naïve animals, this difference did not reach statistical significance (Figure 1B). Similarly, we did not detect differences in promoter-wide methylation at the IFNγ, IL-2 or TNFα promoters in tissue-derived CD8^+^ T cells from SIV-infected compared to SIV-naïve animals likely due to the limited sample size per group (Table 5). Thus, in future studies, it will be essential to obtain longitudinal tissue biopsy samples from the same animal prior to infection and then post-infection or have a larger sample size per group for a well-powered cross-sectional study to assess the impact of epigenetic modifications on tissue CD8^+^ T cells in response to SIV infection.

The tissue analysis, nonetheless, yielded important insights into the role of DNA methylation on CD8^+^ T cell regulation across lymph nodes and intestine within the same animal in both SIV-uninfected and infected groups. Differences in viral burden and immune activation result in distinct immunological milieu in intestinal tissue compared to lymph nodes during HIV/SIV infection [57,59,60,61]. The impact of these factors was evident in the distinct methylation patterns observed in intestinal versus lymph node CD8^+^ T cells, particularly at the IFNγ promoter. At the promoter-wide level, methylation at the IFNγ promoter was significantly lower in intestinal compared to lymph node CD8^+^ T cells in SIV-infected animals, with a similar though non-significant trend observed in SIV uninfected animals (Figure 3A). However, the global DNA methylation levels of total cellular DNA indicated a trend towards an overall repressive state due to higher methylation of intestinal CD8^+^ T cells compared to lymph nodes, independent of SIV status (Figure 1B). Multiple factors likely contribute to the overall higher methylation levels of intestinal CD8^+^ T cell genomic DNA. Firstly, intestinal CD8^+^ T cells in infants exhibit reduced functionality in the early immune development phases [62], and secondly, CD8^+^ T cells progressively become dysregulated due to higher immune activation in the gut [57,59]. In addition, it is important to note that our study assessed methylation signatures from bulk CD8^+^ T cells where the relative proportion and contribution of various CD8^+^ T cell subsets was not determined due to limited samples from infant macaques. Although not tested directly, the relative proportion of polyfunctional, non-recirculating, tissue-resident CD8^+^ T cells and other circulating CD8^+^ T cell subsets at each tissue would impact the methylation signature observed in our study [58,63,64]. Our tissue analysis suggests that the DNA-methylation patterns in CD8^+^ T cells are distinct for each cytokine at different anatomic locations in SIV-infected infant rhesus macaques likely impacted by varying levels of antigen exposure and cellular composition at each tissue site.

This study presents a systematic assessment and characterization of tissue-specific DNA methylation signatures of CD8^+^ T cells both at the total cellular DNA level and at key immunomodulatory cytokines during pediatric SIV infection. This study leveraged unique samples from previously completed infant rhesus macaque studies to elucidate the epigenetic landscape of CD8^+^ T cells across various anatomic sites during infection which is severely understudied in pediatric HIV infections. Despite the low sample numbers, to our knowledge, this is the first study to compare the methylation signature of CD8^+^ T cells from lymph node versus intestinal tissue within the same animal in the infant SIVmac_239/251_ infection model. However, we were unable to perform any additional correlative virologic, transcriptional, or functional assays due to limited sample availability from previously completed infant rhesus macaque studies utilized here. Further studies will be needed to investigate the transcriptional and functional impact of the DNA methylation patterns identified in this study, particularly the role of the differentially methylated CpG sites identified. Methods similar to those used here could be used to evaluate DNA methylation changes at additional immune response genes, including cytokines and chemokines essential for cytolytic activity of CD8^+^ T cells, such as Granzyme B, Perforin, and MIP1β. Overall, this study highlights the importance of assessing cells from both blood and tissue compartments to fully understand the regulation of CD8^+^ T cells and lays the foundation for new research avenues to determine the role of epigenetic modulation, specifically DNA methylation in the regulation of CD8^+^ T cell responses during pediatric HIV infection.

## Figures and Tables

**Table 1:** Study Animals.

Tissue Type/Animal	Age (Wks)	Tissue	SIV Status	Wks PI ^a^	Viremia at the Time of Sampling (RNA Copies/mL)
*PBMC ^b^—Pre- and post-SIV infection*					
RM ^c^ 1	12	PBMC	Negative	—	
	25		Positive	13	1.6 × 10^7^
RM 2	12	PBMC	Negative	—	
	24		Positive	12	1.7 × 10^6^
RM 3	12	PBMC	Negative	—	
	24		Positive	12	2.5 × 10^7^
RM 4	12	PBMC	Negative	—	
	24		Positive	12	7.4 × 10^6^
*Tissues—SIV-uninfected*					
RM 5	12	LN ^d^, INT ^e^	Negative	—	—
RM 6	5	LN, INT	Negative	—	—
RM 7	4	LN, INT	Negative	—	—
RM 8	6	LN, INT	Negative	—	—
*Tissues—SIV-infected*					
RM 9	18	LN, INT	Positive	9	1.4 × 10^8^
RM 10	15	LN, INT	Positive	6	8.7 × 10^7^
RM 11	18	LN, INT	Positive	4	4.0 × 10^7^

^a^ PI = post-infection; ^b^ PBMC = peripheral blood mononuclear cells; ^c^ RM = rhesus macaque; ^d^ LN = lymph nodes; ^e^ INT = intestinal tissues.

**Table 2:** Primer sequences for bisulfite sequencing.

Target (GenBank No.)	Primer ^a^	Sequence (5′-3′) (Position—Input Sequence Used for Primer Design *)	Amplicon Size
IL-2 promoter (EF457241)	F R	AGGTAAAGATATAAAAATGAGAAATATGGATTGG (143–171) ATATAAATAAAATCCCTCATAATTACATTAACCCAC (741–706)	597 bp
IFNγ promoter (AY486428.1)	F R	AATGTGTTTTGTGAATGAGGAGTTAATATTTTATTAGG (412–449) AACTTAACTAATCTTTCTCTTCTAATAACTAATCTTC (763–799)	387 bp
TNFα promoter (AY486430)	F R	AGGAGGAYGGGATTTAATTTTTAGAG (649–674) AAAATCTAAAATTACTTCTCTCCCTCTTAAC (963–992)	343 bp

^a^ F = forward, R = reverse. * see Supplementary Material S2 for primer location.

**Table 3:** Selected regions of cytokine promoters.

Cytokine Promoter	Gen Bank No.	Promoter Region of Interest * (Size)	No. of CpGs in Assessed Promoter Region
IL-2	EF45724	530–1127 (597 bp)	3
IFNγ	AY486428.1	412–799 (387 bp)	3
TNFα	AY486430	649–992 (343 bp)	11

* Promoter Region of Interest- position mentioned with respect to the 1st base pair of promoter sequence for each cytokine per NCBI.

**Table 4:** Predicted transcription factor binding sites in selected regions of cytokine promoters.

Cytokine Promoter	CpG Site Position from TSS ^a^	TF ^b^ Binding Sites Coinciding with or in Close Proximity to CpG Sites
IL-2	−306	C/EBPbeta, XBP-1, GR-beta, Foxp3, HNF-1C, HNF-1A, HNF-1B, C/EBP alpha, POU2F1, POU2F2
	−644	C/EBP beta, GR-alpha, GR-beta, HNF-3A, PR-B, PR-A, MEF-2A
	−702	C/EBP beta, XBP-1, GR-beta, Foxp3, PR-B, PR-A, C/EBP alpha
IFNγ	−183	GR-beta, GR-alpha, TFII-1
	−315	GR-alpha, C/EBP beta, LEF1, NFAT2, NCI/CTF, SRY, TCF-4E, GR, RXR-alpha, NF-1, TCF4
	−410	GR beta, TFIID (T00820), GR-alpha, NFAT2, TFII-1, NFKB, C/EBP beta, STAT4, c-ETS1, STAT1beta, HNF-3alpha, NFAT1, IRF1
TNFα	−172	C/EBP beta, GR-alpha, TFII-1
	−201	C/EBP beta, Foxp3, GR, c-Myb, PR-B, PR-A
	−220	GR-alpha, TFII-1, PPAR-alpha: RXR-alpha, XBP-1, AR, TBP
	−228	GR-alpha, Pax-5, p53, PPAR-alpha: RXR-alpha, ETF, EBF
	−251	C/EBP beta, Foxp3, GR-beta, XBP-1
	−298	GR-alpha, STAT4, c-Ets-1, Elk-1, PU.1, c-Ets2
	−340	C/EBP beta, Foxp3, TFII-1, NFI/CTF
	−342	YY1
	−348	Pax-5, p53, E2F-1, Sp1, WT1
	−417	GR-alpha, TFII-1, PEA3, YY1, MAZ
	−423	GR-alpha, TFII-1, GR-beta, STAT4, c-Ets1

^a^ TSS = transcription Start Site, ^b^ TF = Transcription Factor.

**Table 5:** Cytokine promoter-wide methylation comparison between SIV-uninfected and infected animals in CD8^+^ T cells isolated from lymph node and intestinal tissues.

Tissue Type/Cytokine Promoter	Median Methylation SIV-Naïve Animals (%)	Median Methylation SIV-Infected Animals (%)	95% Confidence Interval of Difference Between Median	*p*-Value ^a^
Lymph node/IL-2 Promoter	82.3	83.1	−8.7 to 7.8	>0.9999
Intestinal tissue/IL-2 Promoter	65.3	66.7	−6.9 to 11.6	>0.9999
Lymph node/IFNγ Promoter	82.6	79.3	−12.8 to 17.2	0.6286
Intestinal tissue/IFNγ Promoter	72.0	71.7	−10.1 to 5.7	0.6286
Lymph node/TNFα Promoter	41.7	49.4	−12.4 to 22.1	0.4000
Intestinal tissue/TNFα Promoter	47.8	53.3	3.8 to 11.8	0.0571

^a^ Mann–Whitney test.

## Data Availability

The original contributions presented in this study are included in the article/Supplementary Material. Raw sequencing data can be made available on request. Further inquiries can be directed to the corresponding author.

## Supplementary Materials

The following supporting information can be downloaded at: https://www.mdpi.com/article/10.3390/v16121839/s1. Supplementary Material S1: Representative flow cytometry plots showing CD8^+^ T cell enrichment following CD8^+^ T cell negative bead sorting of PBMC sample. Supplementary Material S2: Input sequences for cytokine promoters used for bisulfite reduced primer design.
