# Supplementary material for: Tissue-Specific DNA Methylation Changes in CD8+ T Cells During Chronic Simian Immunodeficiency Virus Infection of Infant Rhesus Macaques
Source: Viruses. 2024 Nov 27;16(12):1839. doi: 10.3390/v16121839 (PMC11680437; doi:10.3390/v16121839)
Supplement: Supplementary file 1 [file viruses-16-01839-s001.zip › viruses-3311237-supplementary.pdf]

Supplemental Material 1: Representative flow cytometry plots showing CD8⁺ T cell enrichment following CD8⁺ T cell negative bead sorting of PBMC sample.

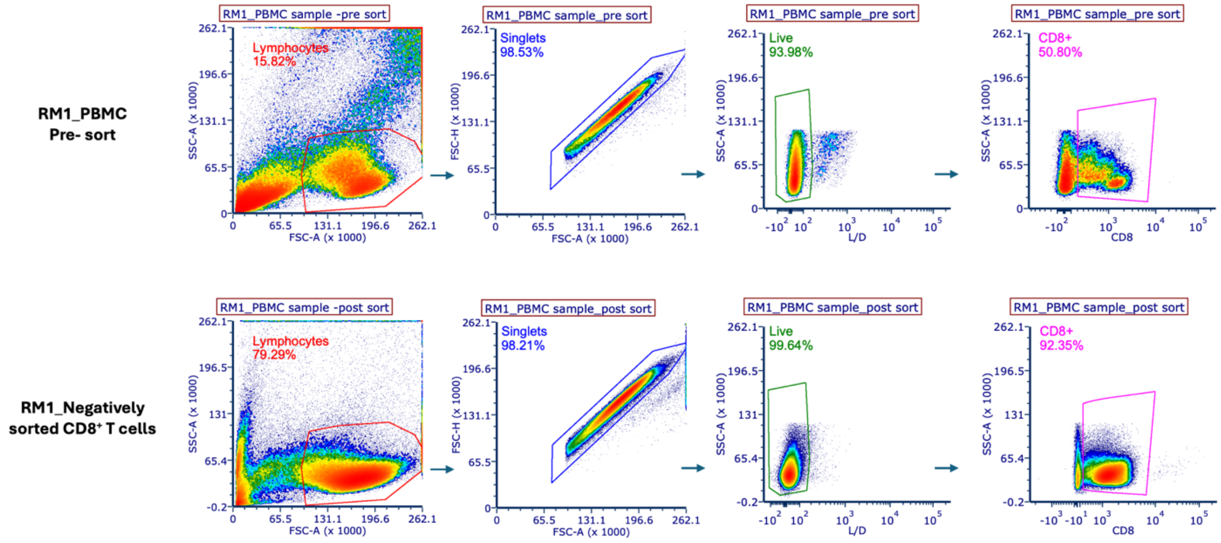

Gating strategy: Lymphocytes → Singlets → Live cells → CD8⁺ cells

Supplemental Material 2: Input sequences for cytokine promoters used for bisulfite reduced primer design
**Note these sequences were bisulfite converted before primer design.*

Key features highlighted:
Red- Transcription Start Site
Blue- CpG sites assessed
Green- Forward Primer
Pink- Reverse Primer

1. **IL2 promoter:** GenBank: EF457241
   https://www.ncbi.nlm.nih.gov/nuccore/133902284

TCAGACAGTTTGTGCATGCTACTGAGGATGAATGAACTCCTACCCTAAATATCCCAATCTGACTAAAAA
AAAGATCGTAACTATTATGGTTACATTCATAGTGTCCCAGGTGATTTAGAGAATAAATAAAAATCCATTAA
AGAGGTAAAGACATAAAAATGAGAAACATGGACTGGTTTACACATAACACATACAAAGTCTATTATAAAA
CTAGCATCAGTATCCTTGAATGCAAACCTTTTTCTGAGTATTTAACAATTGCACCCTTTAAAAAA**CG**TAC
AATAGACATTAAAACACTTAAACAGATATATAAACATTTTAAAATTAAAACAG**CG**TAAAATAGTACCTCAA
GCTCAATAAGCATTTTATGTATTCTAATCTTAGTATTTCTCTAGCTGACATGTAAGAAGCAATCTATCTTAT
TGTATACAATTAGCTCATTGTGTGGATAAAAAGGTAAAACCATTCTGAAACAGGAAACCAAGATACTTCC
TGTTTAATCAACAAATCTAAACATTTATTCTTTTCATCTATTTATTCTTGCTCTTGTCCACCACAATATGCT
ACTCACATGTTCAGTGCAGTTTTATGACAAAGAGAAAATTTTCATGAGTTACTTTTGTATCCCCACCCC
CTTAAAGAAAGGAGGAAAAACTGTTTCATACAGAAGG**CG**TTAATTGCATGAATTAGAGCTATCACCTAA
GTGTGGGCTAATGTAACCATGAGGGATTTCACCTACATCCATTCAGTCAGTCTTTGGGGGTTTAAAGA
AATTCCAAAGAGTCATCAGAAGAGGAAAAATGAAGGTAATGTTTTTTCAGACAGGTAAAGTCTTTGAAA
ATATGTGTAATATGTAAAACATTTTGACACCCCCATAATATTTTTCCAGAATTAACAGTATAAATTGCATCT
CTTGTTCAAGAGTTCCCTATCACTCTCTTTAATCACTACTCACAGTAACCTCAACTGCTGCCACA**ATG**T
ACAGGATGCAACTCCTGTC

2. **IFNγ promoter:** GenBank: AY486428.1
   https://www.ncbi.nlm.nih.gov/nuccore/AY486428.1

AAATTTTAAAAGGCTCCCCCTTTGTAAAGGTTTGAGAGGCCCTAGAATTTCCTTTTTCACTTGTTCCCA
ACCACAAGCAAATGATCAATGTGCTTTGTGAATGAGGAGTCAACATTTTACCAGGGTGAAGGGGGA
GGTG**CG**AAAAAATTTCCAGTCCTTGAATGGTGTGAAGTAAAAGTGCCTTCAAAGAATCCCACCAGAAT
GGCACAGGTGGGCATAATGGGTCTGTCTCAT**CG**TCAAAGGACCCAAGGAGTCTAAAGGAAACTCTAA
CTACAACACCCAAATGCCACAAAACCTTAGTTATTAATACAAACTAGCATCCCTGCCTATCTGTCACCAT
CTCATCTTAAAAAACTTGTGAAAATA**CG**TAATCCTGAGGAGACTTCAATTAGGTATAAATACCAGCAGCC
AAAGGAGGTGCAGCACATTGTTCTGATCATCTGAAGATCAGCTATTAGAAGAGAAAGATCAGTTAAGC
CCTTTGGACCCGATCAACTTGATACAAGAACTTCTGATTTCAACTTCTTTGGCTTAACTCTCTCTGAAA
CG**ATG**

3. **TNFα promoter:** GenBank: AY486430
   https://www.ncbi.nlm.nih.gov/nuccore/AY486430.1

CTTTGAGGGGCAGGAGGACGGGATTCAACCTCCAGAGTCCCACACACAAATCAGTCAGAGGCCCAG
AAGACCCCCCT**CG**GAAT**CG**GAGCAGGGATGGTGGGGAGAGTGAGGAGTATCCTTGATGCTTGGGTG
TCCCCAACTTTCCAAATCCC**CG**CCCC**CGCG**ATGGAGAAGAAACCAAGACAGAAGGTGCAGGGCCCA
CTAC**CG**CTTCCTCCAGATGAGCTCATGGGTTTCTCCACCAAGGAAGTTTTC**CG**CTGGTTGAATGATTC

TATCCC**CG**CCCTCT**CG**CCCCAGGGACATATAAA**CG**CAGTTGTTTGCACACCCAGCCAGCAGA**CG**CTC
CCTCAGCAAGGACAGCAGAGGACCAGCTAAGAGGGAGAGAAGCAACTCCAGACCCCAACTGAAAAA
AAACCCTCAGACGCCACATCCCCAGACAAGCTGCCAAGCGGGTTCTCTCCCTCTCACACACTGACC
CAGGGCTCCACCCTCTCTTCACTGGAAAGGACACC**ATG**
